# Identification of a robust gene signature that predicts breast cancer outcome in independent data sets

**DOI:** 10.1186/1471-2407-7-61

**Published:** 2007-04-11

**Authors:** James E Korkola, Ekaterina Blaveri, Sandy DeVries, Dan H Moore, E Shelley Hwang, Yunn-Yi Chen, Anne LH Estep, Karen L Chew, Ronald H Jensen, Frederic M Waldman

**Affiliations:** 1 Comprehensive Cancer Center, University of California, San Francisco, San Francisco 94143, CA, USA; 2 Department of Laboratory Medicine University of California, San Francisco, San Francisco 94143, CA USA; 3 Department of Surgery University of California, San Francisco, San Francisco 94143, CA, USA; 4 Department of Pathology, University of California San Francisco, San Francisco, 94143, CA, USA; 5 Department of Cell Biology, Memorial Sloan Kettering Comprehensive Cancer Center, 10021, NY, USA

## Abstract

**Background:**

Breast cancer is a heterogeneous disease, presenting with a wide range of histologic, clinical, and genetic features. Microarray technology has shown promise in predicting outcome in these patients.

**Methods:**

We profiled 162 breast tumors using expression microarrays to stratify tumors based on gene expression. A subset of 55 tumors with extensive follow-up was used to identify gene sets that predicted outcome. The predictive gene set was further tested in previously published data sets.

**Results:**

We used different statistical methods to identify three gene sets associated with disease free survival. A fourth gene set, consisting of 21 genes in common to all three sets, also had the ability to predict patient outcome. To validate the predictive utility of this derived gene set, it was tested in two published data sets from other groups. This gene set resulted in significant separation of patients on the basis of survival in these data sets, correctly predicting outcome in 62–65% of patients. By comparing outcome prediction within subgroups based on ER status, grade, and nodal status, we found that our gene set was most effective in predicting outcome in ER positive and node negative tumors.

**Conclusion:**

This robust gene selection with extensive validation has identified a predictive gene set that may have clinical utility for outcome prediction in breast cancer patients.

## Background

The application of expression microarray profiling technology promises to change both our understanding of tumor biology and our clinical practices. Expression arrays have proven useful in a variety of fields, allowing us to examine gene expression dynamics during complex processes such as growth and proliferation [1], as well as to identify gene function by expression patterns [2]. In particular, cancer researchers have made use of this technology to distinguish distinct subsets of cancer, predict patient outcome, and identify genes with clinical relevance [3-8]. Breast cancer has been one of the diseases most extensively studied with microarrays.

Breast cancer is a heterogeneous disease [9], making it an ideal disease to study using microarrays since different expression patterns can be identified within distinct tumor groups. Expression array studies of breast cancer have identified genes associated with histology [6], grade [10], and estrogen receptor (ER) status [5,11]. Perhaps the most important contribution of microarrays to breast cancer research has been the identification of gene sets that are predictive of patient outcome in breast cancer [5,10-14], with an accuracy that surpasses traditional predictive factors. Indeed, it has been proposed that such gene sets should be employed in clinical practice to aid in decision making for patients. However, the genes identified in these predictive sets have shown little overlap, making it unclear which genes or gene sets are best. We report gene expression profiling on a diverse panel of breast tumors using both unsupervised and supervised methods to distinguish clinically relevant subgroups on the basis of gene expression. Our robust gene selection and validation demonstrates that predictive gene sets derived from expression microarray data can perform as independent predictive markers, and thus may have great clinical utility.

## Methods

### Samples

Fresh frozen breast tumors were collected from patients treated at UCSF and California Pacific Medical Centers after institutional review board approval. Randomly selected frozen tumor blocks were trimmed to ensure that a minimum of 70% of the remaining cells present were tumor. A total of 140 invasive ductal tumors, 17 invasive lobular tumors, 4 DCIS, 1 inflammatory, and 8 normal breast samples from reduction mammoplasties were analyzed. There were 4 DCIS, 35 Stage I, 80 Stage II, 16 Stage III, 6 Stage IV tumors, and 21 with unknown stage. ER negative tumors comprised 29% of the samples, and 45% of the samples were node negative. Full clinical information can be found as an additional file (see additional file 1). A subset of 55 samples was chosen for outcome analysis, which consisted of a Good Outcome (GO) group of tumors that had at least 7 years disease free survival, and a Poor Outcome (PO) group of samples that were never disease free or had distant relapse of disease within 7 years.

### RNA isolation

RNA was isolated as described elsewhere [6]. Briefly, frozen sections were placed directly in Trizol reagent (Invitrogen, Carlsbad, CA), homogenized, and RNA was isolated using the manufacturer's protocol. RNA was quantified based on absorbance at 260 nm. Quality of the RNA was assured by measuring 260/280 ratios, and reviewing integrity on agarose denaturing gels. Some samples that had been stored in formamide were further purified through RNeasy columns (QIAGEN, Valencia, CA) according to the manufacturer's protocol. We saw no effects on expression as a result of this storage or extraction compared to samples that were not processed in this manner. A mixture of equal amounts of RNA from the following cell lines, all available from ATCC, was used as a common hybridization reference sample: SW872, WM115, NTERA2, MCF7, HEPG2, MOLT4, Hs578t, HL60, OVCAR3, COLO205, and RPMI 8226. The same preparation of reference pool RNA was used for all hybridizations.

### Expression microarray preparation

Preparation of the microarrays used in this study is described elsewhere [6]. Briefly, clones from the Research Genetics clone set (Research Genetics, Huntsville AL) were PCR amplified using universal primers, band size was verified, clones were precipitated, dissolved in 3 × SSC, and printed on poly-L-lysine (Sigma, St Louis, MO) coated slides. Microarrays consisted of 32 subarrays, each 18 rows by 18 columns, for a total of 10,368 spots. Slides were stored under vacuum. Post-processing was done as described elsewhere [6]. Microarrays from 6 different print runs (using the same clone preparations from the same plates) were used in this study.

### Labeling and hybridization

Labeling was done as described elsewhere [6]. Briefly, 5–10 μg of RNA that was DNase I treated using DNAfree reagent (Ambion, Austin, TX) was reverse transcribed with superscript II reverse transcriptase (Invitrogen) in the presence of amino-allyl modified dUTP (Sigma) using random hexamers (Invitrogen) and Oligo dT primers (Invitrogen). The cDNA product was coupled to free Cy3 dye (Amersham, Piscataway NJ), mixed with a Cy5 labeled reference, Cot-1 DNA (Invitrogen), and tRNA (Invitrogen) in 25 mM HEPES, 3 × SSC, and 0.03% SDS. Hybridization was performed at 65°C overnight in a HybChamber (Gene Machines, San Carlos, CA). High stringency washes were in 2 × SSC, 1% SDS at 65°C for 10 minutes, 2 × SSC at room temperature for 10 minutes, and 0.2 × SSC at room temperature for 10 minutes [15]. Slides were briefly rinsed in deionized water and dried with compressed air. Slides were imaged on an Axon 9000B scanner (Axon Instruments, Union City, CA).

### Statistical analysis

All images were analyzed using GenePix pro v3.06 (Axon Instruments). Raw .gpr files and the derived log_2 _ratios are available as additional files. Data were corrected by subarray (print tip) median centering and LOWESS smoothing using the Bioconductor R software package [16]. The LOWESS corrected data is available as an additional file (see Additional file 2). Data were normalized by print prior to unsupervised clustering using Eisen's Cluster and TreeView [17] to avoid print specific effects (see Additional file 3). For clustering, clones were chosen based on the entire tumor sample set; they were accepted if they were present in at least 80% of cases and showed a log_2 _test over reference ratio [log_2 _(t/r)] of less than -2 or greater than 2 in at least one case, resulting in a total of ~4,000 genes. All genes and arrays were median centered and normalized. Samples were divided into the GO and PO groups as described above for outcome prediction. Prediction Analysis for Microarrays (PAM) [18], Significance Analysis for Microarrays (SAM) [19], and a correlation based technique [12] with a threshold set at 0.85 were employed to identify genes associated with good outcome and to classify samples on the basis of gene expression. All genes identified in the predictive sets were sequence validated, with genes failing sequence verification excluded from the final analysis. If multiple copies of the same gene were identified, they were each treated as an individual gene (i.e. replicate clones were not averaged). We have previously validated expression data derived from these microarrays using real-time quantitative PCR, which showed complete agreement with the microarray data [6]. Classification of tumors for all gene sets was performed using a leave one out cross validation analysis based on correlation of each tumor's gene expression with the means of the remaining "good" or "bad" outcome tumors. To accurately estimate classification rates, gene re-sampling was performed during each round of cross-validation [20]. Kaplan-Meier survival curves were generated based on the good and predicted poor prognosis groups using the survival package within Bioconductor R.

## Results

### Unsupervised clustering of breast samples

**Figure 1:**
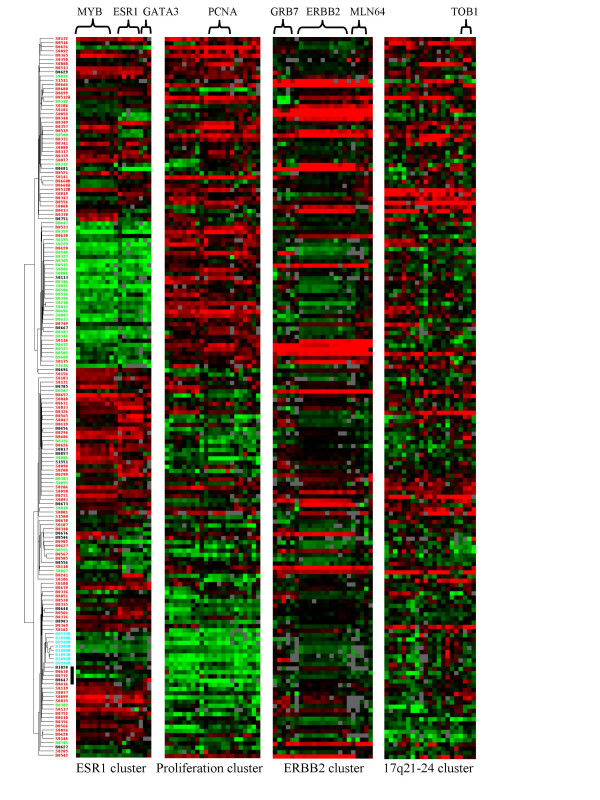
Unsupervised hierarchical clustering of 162 breast tumor samples based on ~4,000 genes with greatest variation. Tumor samples (left) are color coded according to immunohistochemical staining for estrogen receptor (ER negative, green; ER positive, red; reduction mammoplasties, cyan; ER status unknown, black). Gene clusters (top) are those related to estrogen receptor (ESR1), proliferation-associated genes, ERBB2 related genes, and genes found in a common chromosomal region of 17q21-24 (red represents high relative expression, green low relative expression). The black bar indicates samples that clustered tightly with normal specimens.

Hierarchical clustering of the 162 tumor and 8 normal samples resulted in the formation of distinct tumor groups based on gene clustering, as has been previously described [12]. The most prominent tumor group was related to estrogen receptor(*ESR1*) expression (Figure 1). As expected, there was a strong correlation between *ESR1 *expression levels and ER status by immunohistochemistry (IHC). The average expression level of the *ESR1 *gene in tumors that were ER negative by IHC was 0.66 (log_2 _t/r), while ER positive tumors had an average *ESR1 *expression level of 3.06. For the quartile of samples with the lowest *ESR1 *expression levels, 86% were classified as ER negative by IHC, while 97% of the quartile of samples with the highest *ESR1 *expression levels were ER positive. Genes that were correlated with *ESR1 *expression included *GATA-3, β-MYB, BCL-2*, and *DPP6*, all of which have previously been shown to be co-expressed [12].

A set of 5 tumor samples clustered with the normal samples from reduction mammoplasties (Figure 1). This group was characterized by low levels of expression of genes associated with proliferation, such as *CCNA2, PCNA, GART, CDC2*, and a number of histone genes, all of which clustered together. The central part of this proliferation cluster, including *PCNA*, is shown in Figure 1. Expression of these genes was high in the ER negative tumor group as well as a group of ER positive tumors (Figure 1).

A cluster of genes associated with *ERBB2 *expression was also evident in the unsupervised clustering heatmap (Figure 1). In addition to *ERBB2*, the *MLN64, PSMB3, PIP5K2B*, and *GRB7 *genes, all of which map to 17q12, were found in this cluster as has been previously described [21]. There was another related but distinct cluster of genes that map to a region proximal to the ERBB2 amplicon. Included in this cluster were genes such as *COX11*,*TRAP240, PHB, UGTREL1, SUPT4H1, AKAP1, COIL, PSMC5, GK001, RPS6KB1, CLTC, PSMC5, AKAP1, COIL, APPBP2, DDX5, FALZ, NME1, NME2*, and *TOB1*, and the ESTs AA495944 and AA458968, all of which map in the region 17q21.33-17q24.2. The treeview-cluster files for all 4,000 genes used for the unsupervised analysis are available as additional files (see Additional file 4).

Unlike ER status, other clinical parameters such as nodal status, stage, and grade did not show strong correlation with any of the tumor clusters formed by unsupervised hierarchical clustering.

### Outcome prediction

To identify genes associated with outcome, tumor samples from patients with seven year disease free survival versus patients having recurrence within seven years were compared. Seven years was chosen as a balance between long follow up length and a sufficient number of tumors for analysis. The average follow-up time (disease free survival) in the GO group (34 samples) was 9.82 years, while the average disease free survival time in the PO group (21 samples) was 2.78 years. Kaplan-Meier analysis using the clinical parameters ER status, stage, and grade did not result in significant associations with survival in this data set, presumably due to sample selection criteria. However, nodal status showed a significant correlation with disease free survival.

There are a number of supervised methods that utilize gene expression data for the identification of genes and tumor classification. Several of these methods were used to identify predictive gene sets (see Additional File 5). The first method used the PAM software package [18] to identify a set of 142 genes that predicted class (PO vs. GO). The leave one-out-cross validated classification rate performed with gene re-sampling was 71% with this gene set (76% of GO cases, 62% of PO cases). Interestingly, although ER status was not a predictor of outcome in these tumors, two copies of *ESR1 *and multiple copies of the β-*myb *gene, which is correlated with *ESR1 *expression, were included in this predictive gene set. There were also multiple copies of *ZNF217*, *LTF*, *DHRS4*, *NFIC*, *GNAS1*, and *NAT1 *present in this gene set.

A second approach, which used a correlation based method similar to the one used by van't Veer et al. [12], identified a set of 49 genes that correctly classified 75% of the samples (79% of GO cases, 67% of PO cases). There was a strong overlap between this gene set and that from PAM, with 40 out of 49 genes included in the PAM set.

We also used an approach to identify a set of 49 genes that were differentially expressed between GO and PO tumors using SAM. The genes in this set had an estimated false discovery rate of 10% (less than 5 genes expected to be false positive). Of the 49 genes, 46 were also present in the gene list derived from PAM analysis. Since this method is not a classification technique, we did not test the predictive utility of these genes in the training set.

**Figure 2:**
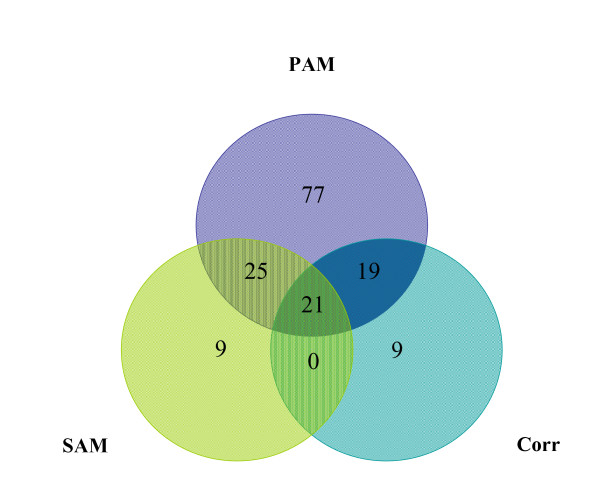
Relationship between PAM, SAM, Correlation, and overlapping gene sets. Degree of overlap between the 142 (PAM), 49 (SAM), and 49 (correlation) gene sets which results in the 21 gene set.

We were interested in developing a clinically relevant predictive gene set, which necessitated a reduction in the number of genes in the predictor, since a large number of genes is prohibitive for clinical assays such as quantitative rt-PCR. Thus, a fourth gene set was constructed consisting of genes that overlapped from the three earlier gene sets, resulting in a set of 21 genes. We used the leave-one-out cross-validation approach with gene re-sampling during each round of validation to classify the samples based on these 21 genes. This gene set correctly classified 69% of the tumors (74% of GO cases, 62% of PO cases) using the leave-one-out cross- validation approach. The relationship between the various gene sets is shown in Figure 2.

The cross-validated classification rates using the three predictive gene sets ranged from 69–75% in the training set. It should be noted that using most traditional clinical outcome predictors, even lower rates were achieved in this tumor set. For example, ER status correctly predicted outcome in 64% of cases. Only nodal status was comparable, correctly predicting outcome in 71% of cases.

**Figure 3:**
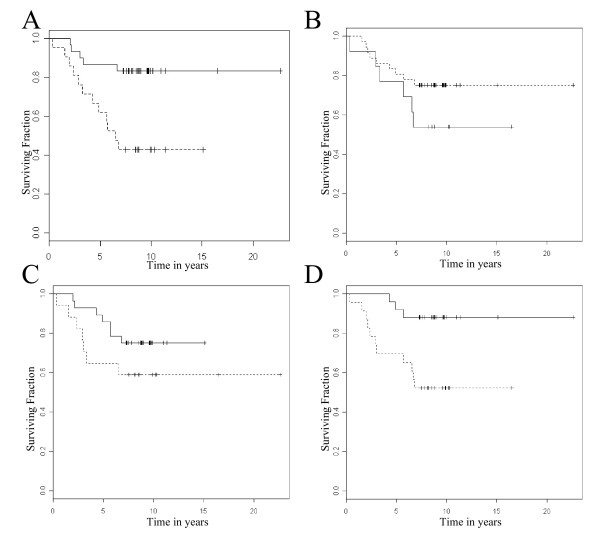
Kaplan-Meier survival curves for UCSF breast tumors based on our predictive gene sets. Surviving fraction of predicted good (solid lines) and poor (dashed lines) groups is shown. A) separation based on 21 gene set (*P *< 0.0001). B) separation based on ER positive (dashed line) vs. ER negative (solid line) (*P *= 0.18). C) separation based on grade 1+2 (solid line) vs. grade 3 (dashed line) (*P *= 0.19). D) separation based on node positive (dashed line) vs. node negative (solid line) (*P *= 0.005).

Kaplan-Meier survival curves were constructed based on tumor classification using the leave-one-out classification with gene re-sampling for each of the three predictive gene sets. The results of the classification and *P *values from the Kaplan-Meier survival curves for each gene set are summarized in Table 1. The disease free survival of the predicted good group was significantly better than the predicted poor group for each gene set (see Figure 3A for survival curve for the 21 gene classifier). In contrast, for ER (Fig. 3B), grade (Fig. 3C), and nodal status (Fig. 3D), only nodal status showed significant differences in disease free survival times.

To test whether classification was being driven by phenotype or treatment modality, we examined tumor classification within subtypes. For phenotype analysis, tumors were stratified by ER status, nodal status, and Grade. ER positive, node negative, and low grade tumors were classified most successfully (Table 2). For treatment modality, tumors were stratified into those that were treated with radiation, chemotherapy, or hormonal therapy compared to tumors that were untreated. In general, the patients who did not receive radiation and/or chemotherapy were classified more successfully, while those who received hormonal therapy showed mixed results (Table 3).

### Functional annotation of genes

To determine if there were any pathways that were over-represented in the 21 gene set, we examined their gene ontology categories. Two genes (*DHRS4*, *GALK1*, are implicated in metabolism, two are transcription factors (*ZNF217*, *TAL1*), three have roles in cell cycle regulation (*MAD2L1*, *YWHAQ*, *BRCA2*), two in transport (*NGB, SLC9A3*), two in development or differentiation (*CKTSF1B1*, *GMFB*), and five have been implicated in signaling (*LTBR*, *NRG1*, *KIFAP3*, *CD3D*, *GNAS*).

### Comparison to published predictive gene sets

Since we did not have an adequate number of additional specimens with long term follow-up to test our predictive gene set, we turned to other published data sets. There have been several other predictive gene sets for breast cancer that have been reported [10,12,14,22]. We compared the predictive genes in several of these sets to our predictive gene lists. Sorlie et al. identified a set of 534 genes (the "intrinsic" gene set) that separated breast tumors into distinct subtypes which predicted outcome [11] and subsequently showed that this gene set was predictive in several other breast tumor data sets [14]. There were 402 genes in their intrinsic set that were also present in our data set. PAM was used to classify their tumors based on these 402 genes, and our tumors were fit to the different subtypes. Forty three of our tumors were classified as Luminal Type A, zero classified as Luminal Type B, 5 ERBB2 positive, 4 Basal-like, and 3 Normal-like. The tumor groups showed significantly different survival, with *P *= 0.019 (Hazard Ratio = 1.17).

There were 11 genes in common between our data set and that of Sotiriou [10], including two genes involved in guanine nucleotide binding (*GNAS1*, *GNAL*), the insulin receptor, and the MAD2-like 1 homolog. There were 4 genes in common between the van't Veer predictive set [12] and ours, including survivin (*BIRC5*) and *STK15*. The only gene common to all three sets was *MAD2L1*. More recently, a smaller 21 gene set has been developed [22], based on a combination of genes selected from published microarray work and traditional clinical markers. Of these 21 genes, 16 have predictive utility, while the remaining 5 are reference genes. Our gene sets had 7 genes in common with the 16 predictive genes, namely *STK15*, *BIRC5*, *MYBL2*, *MMP11*, *ERBB2*, *GSTM1*, and *ESR1*.

**Figure 4:**
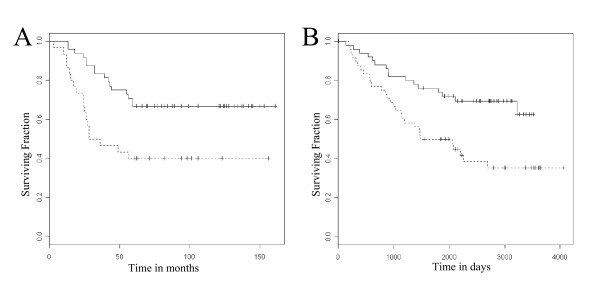
Kaplan-Meier survival curves based on the 21 gene set in independent data sets. A) van't Veer et al. data set; B) Sotiriou et al. data set. Surviving fraction for predicted good (solid lines) and poor (dashed lines) groups in the validation tumor sets are shown (*P *< 0.0005).

We tested the performance of our 21 gene set in the tumor sets of van't Veer et al. and Sotiriou et al. using PAM classification. The gene set classified 65% of the specimens correctly in the van't Veer tumor set, and resulted in significant differences (*P *< 0.005, Hazard Ratio = 2.76) in survival between good and poor prognosis tumor groups in Kaplan Meier analysis (Figure 4A). The performance of the gene sets in the Sotiriou tumor set was similar, with a classification rate of 62%. As with the van't Veer data set, this resulted in significant differences (*P *< 0.01, Hazard Ratio = 2.37) in survival of the good and poor prognosis groups (Figure 4B).

As a further validation, we compared the performance of the van't Veer predictive gene set and the Sotiriou predictive gene set in our tumors. We were able to identify 89 genes (115 clones) that were present on our microarrays from the full set of 231 predictive genes from van't Veer et al. [12]. These genes correctly classified 64% of our tumor samples, but did not result in significant separation in Kaplan Meier analysis (*P *= 0.108, Hazard Ratio = 2.00). A subset consisting of 70 clones (68 genes) was found to be optimal by van't Veer et al. for outcome prediction. We were able to identify only 22 genes (25 clones) from our data set that corresponded to genes from their 68 gene set. This gene set classified 73% of the UCSF samples correctly, but did not result in significant separation of GP and PP groups by Kaplan-Meier survival analysis (*P *= 0.14, Hazard Ratio = 1.99), likely due to almost all samples being classified as belonging to the good outcome group. Sotiriou et al. identified a predictive gene set of 424 genes (485 clones) derived using a panel of breast tumors with a variety of stages [10]. We were able to identify 318 genes (452 clones) from their predictive gene set that were present on our arrays. This gene set classified 64% of the UCSF samples correctly, but did not achieve significance in separating good and poor prognosis patients in our data set (*P *= 0.10).

## Discussion

Expression microarray technology promises to change phenotypic characterization of tumors, leading to better diagnosis, prognosis, and ultimately treatment of cancer. Expression profiling has been used to identify predictive gene sets in a diverse set of cancers, including lymphomas [7,23,24], prostate cancer [25], and breast cancer [3,5,10-12,26]. These gene signatures are more robust than individual prognostic genes that have been identified. Unlike single gene predictors, gene sets are less likely to be influenced by variation in expression of one or two genes when classifying tumor specimens, since they use the entire set of genes to classify samples, not just one or two. Furthermore, tumors that are indistinguishable using traditional clinical parameters can be classified into good and poor outcome groups using predictive gene sets, and thus these sets may have the ability to outperform the traditional markers. However, the identities of genes within classifiers differ widely even for the same tumor types, despite the fact that the association of specific expression patterns with tumor phenotypes is clear. Robust gene selection techniques and extensive validation are required to identify the gene sets which best predict patient outcome.

We identified multiple gene sets based on several predictive models, then validated them with an independent tumor set. We used three methods to identify potential predictive gene sets, all of which resulted in significant differences in survival of the predicted good and poor prognosis groups in both the test and validation tumor sets. We reasoned that the most robust gene set was one which consisted of all of the overlapping genes from the three selection methods, consisting of 21 genes. As with the other 3 gene sets, this predictive set resulted in significant differences in survival for the good and poor prognosis groups.

Multivariate analysis of the 21 gene set indicated that it was not an independent prognostic marker when used in combination with nodal status or stage for prediction of outcome in our tumor set (data not shown). However, because of the small number of events in the data set, we had limited power for determining the predictive ability of the gene set in a multivariate model.

We tested the performance of our gene sets in the tumor sets of van't Veer et al. and Sotiriou et al. There are several caveats with such an approach. First, it has been observed that there is often disagreement between microarray results profiled on different platforms [22,27]. Part of this may be attributable to different probe regions represented (i.e., splice variants), different hybridization, washing, imaging, and data normalization and analysis methods, and the use of different (or no) reference samples. Another problem comparing microarray results is that there are often genes missing from one platform that are identified as being predictive using the second gene set. Obviously, these cannot be included in the comparative analysis, so the model is weakened by the absence of core predictive genes. Finally, as in our data, there is a difference in the way tumors were selected, so that genes that are predictive in a diverse set of tumors such as ours may not perform as well in a more homogeneous tumor set such as that of van't Veer. Indeed, we found that the performance of our gene sets was not as good in the tumor sets of van't Veer et al. and Sotiriou et al., but still resulted in significant differences in survial with Kaplan Meier analysis. Furthermore, the performance of our predictive gene sets was comparable to those of van't Veer and Sotiriou in our tumor sets, although none of their gene sets resulted in significant differences in survival with Kaplan-Meier analysis when used on our tumor set. In particular, the failure of the van't Veer predictive set to correctly classify our tumors likely reflects the fact that more than half of their predictive genes were missing from our microarrays.

It worth noting that each predictive gene set performed best within the tumor set from which it was derived. This is not surprising, since there is an inherent bias introduced by testing genes on the tumors from which the genes were selected. Thus, it is important to validate the predictive utility of genes in independent data sets, which requires making such data sets publicly available. Ideally, a common platform will come into use so that investigators will be able to make easy comparisons between experiments, without having to exclude potentially important genes from their validation analyses. This would also allow a comprehensive meta-analysis of genes in common to the predictive gene lists under investigation to identify those with the strongest prediction of breast cancer outcome. To date, the best solution to these problems has been the development of more rigorous statistical techniques and better laboratory practices, which improve concordance in cross-platform comparisons [28].

One striking observation is the minimal overlap between genes in the predictive gene sets developed by us and those of van't Veer et al. and Sotiriou et al. Sotiriou reported that their predictive gene set had 15 genes in common with the van't Veer set [10]. We had 4 genes in common with the van't Veer predictive gene set, and 11 genes in common with the Sotiriou predictive gene set. All three sets had 1 gene in common (*MAD2L1*). However, it is interesting to note that of 16 predictive genes utilized in a more recent study [22], seven were also found to be predictive in at least one of our gene sets. While the overlapping genes are likely to be important in outcome prediction, it may be inappropriate to focus entirely on these genes. The comparisons between the three different microarrays are by no means comprehensive. For example, almost 100 of the genes identified by van't Veer as being predictive were not present on our arrays. Thus, the lack of overlap between the predictive sets may reflect the lack of overlap of the arrays in general. A recent study examining poor overlap in predictive gene sets derived from separate studies in breast cancer [29] indicated that the poor gene overlap was due to the fact that a number of genes showed correlation with survival, but that these associations vary greatly between subsets of patients.

Interestingly, while the overlap between the gene sets is minimal, there is some evidence that similar families of genes are found within the different classifiers. Thus, it may be possible to identify a set of genes, each of which is interchangeable with the other members of that gene set with respect to their predictive abilities. For example, we found that high levels of *ESR1 *and the *MYB *gene, which has been shown to be coordinately expressed with *ESR1 *[5,26], were both predictive of outcome in our data set. Neither the van't Veer nor Sotiriou predictive gene sets contained these genes, but the predictive set of Sotiriou et al. [10] included *GATA3*, a gene which has also been shown to be coordinately expressed with *ESR1*. Thus, it is possible that *ESR1, GATA3*, or *MYB *may be surrogates for one another in predicting outcome.

Unsupervised clustering of the entire data set resulted in separation of the tumors based primarily on their ER status. This has been observed previously by several groups [5,26], and is a strong factor driving unsupervised clustering in breast cancer. There was a strong correlation between *ESR1 *expression and ER status as measured by IHC. While promoter methylation and chromatin condensation of *ESR1 *gene seems to be the predominant mechanism for ablation of ER protein expression [30], the finding that a number of ER negative tumors had higher than average levels of *ESR1 *gene expression suggests that some tumors may be ER negative due to post-transcriptional events.

The putative transcription factor *ZNF217 *was identified within our predictive gene sets, and overexpression was associated with poor outcome in our breast cancer patients. This gene was originally identified as a potential target oncogene from the 20q13 region, which is commonly amplified in breast and other cancers and has been associated with poor prognosis [31,32]. Subsequent analysis of *ZNF217 *has shown that it is capable of immortalizing human mammary epithelial cells [33]. Interestingly, in addition to *ZNF217*, we observed that high levels of expression of several genes from the 20q13 region were associated with poor prognosis in the breast cancer patients. Included in this region were *STK15*, which has recently been suggested to be a candidate low-penetrance tumor susceptibility gene in breast cancer [34] and *MYBL2*, which has been shown to be overexpressed along with *STK15 *and *ZNF217 *in prostate cancer [35]. Overexpression of *STK15 *and *MYBL2 *was found to be associated with metastases in these prostate tumors [35], and in our data set high levels of expression of all three genes were associated with poor prognosis. Interestingly, both *MYBL2 *and *STK15 *were found to be predictive by Paik et al. [22], and *STK15 *was found to be predictive by van't Veer et al[12].

Generation of predictive gene sets has been done primarily in mixed tumor sets (e.g., ones that include both ER negative and ER positive, and with the exception of van't Veer et al, in node negative and node positive samples). By examining the classification rate within the tumor subgroups, it is evident that our predictive gene sets tend to perform better in ER positive, Stage I and II, and node negative tumors. Similarly, the gene prediction classifier tended to perform better in patients who did not receive chemotherapy or radiation. Together, these results are encouraging, since these tumors tend to have better prognosis in general and thus it is difficult to determine which patients are at highest risk. Future studies to identify predictive gene sets within clinically homogeneous subgroups of breast cancer may further improve outcome prediction based on genetic signatures.

## Conclusion

Our gene sets may have potential clinical utility since they demonstrated predictive ability in both our breast cancer tumor sets and, to a lesser extent, in two independent tumor sets. At this time, however, it is unclear how predictive gene sets will be applied in clinical practice. Tests relying on diagnostic expression chips would likely require fresh frozen material from which intact RNA could be extracted, which could be a limitation. PCR based tests may be possible from archival paraffin material, but would likely be limited to small predictive gene sets similar to the 21 gene set we have identified. Diagnostic breast cancer tests based on expression are already being offered commercially, although it is unclear which specific sets are superior, due to minimal gene overlap between predictive sets. Indeed, the ideal diagnostic test sets may be composed of genes with the best predictive ability from several different gene sets. Clinical implementation of such a gene set would require regulatory and other issues to be resolved, including finalization of an ideal diagnostic set and use of a common platform to allow standardization, manufacturing, and quality control. Furthermore, while these diagnostic genes may accurately predict which patients will fare better, we still have not determined how to best treat patients with a poor prognosis signature. These complex issues will need to be fully addressed in order to successfully apply these gene classifiers to clinical practice.

## Abbreviations

estrogen receptor (ER); Good Outcome (GO); Poor Outcome (PO); log_2 _test over reference ratio [log_2 _(t/r)]; Prediction Analysis for Microarrays (PAM); Significance Analysis for Microarrays (SAM); immunohistochemistry (IHC).

## Competing interests

The author(s) declare that they have no competing interests.

## Authors' contributions

JK participated in the study design, performed most of the laboratory experiments, including data and statistical analyses, and drafted the manuscript. EB assisted in microarray preparation and performed the sequence validation for significant clones. SD was involved in microarray preparation, sample selection and RNA isolation, and management of clinical information for the samples. DM assisted in statistical analysis. ESH was involved in study design and clinical review of data. Y-YC reviewed slides for pathological determination of tumor content and histologic classification. AH assisted in microarray preparation and performed post-processing of microarrays. KC was involved in pathologic review of tumor specimens and clinical database management. RJ was involved in overseeing microarray preparation and quality control. FW conceived of the study, participated in study design and coordination, statistical analyses, and drafting of the manuscript.

## Pre-publication history

The pre-publication history for this paper can be accessed here:



## Supplementary Material

Additional File 1 One excel file containing clinical data for the full set of breast tumors used in this study. Available under clinical information for all cases (and outcome cases) links [36]. Click here for file

Additional File 2 Excel file containing lowess corrected ratio data. Available under expression ratio values for all cases (and outcome cases) [36]. Click here for file

Additional File 3 Excel file containing the median centered, lowess corrected data with print normalization. Available under expression ratio values for all cases after centering and normalization [36]. Click here for file

Additional File 4 Treeview and cluster files for viewing of treeview images (.cdt, .atr, and .gtr files) Available under hierarchical clustering files [36]. Click here for file

Additional File 5 One excel file listing the gene sets derived from PAM, SAM, a correlation based technique, and the overlapping gene list. Available under cDNA clones and gene identifiers in UCSF prediction sets [36]. Click here for file

## Figures and Tables

**Table 1:** Classification Rates and Survival Differences for identified gene sets

**Gene Set**	**Genes (N)**^**1**^	**Classification Rate**^**2**^	**Survival *P ***^**3**^
PAM	142	71%	< 0.00001
Correlation	49	75%	< 0.00001
Overlap	21	69%	< 0.005

^1 ^Number of genes identified from each of the predictive sets

^2 ^Leave-one-out cross-validation classification rate using gene re-sampling based on known outcome of 55 tumor samples

^3 ^Kaplan-Meier log rank survival difference using disease-free survival time based on good and poor predicted groups from leave-one-out classification

**Table 2:** Classification Rates by Clinical Subtype^1^

**Gene Set**	**ER Neg N = 15**	**ER Pos N = 40**	**Node Neg N = 25**	**Node Pos N = 29**	**Grade 1 N = 7**	**Grade 2 N = 24**	**Grade 3 N = 20**
PAM	53%	78%	88%	55%	100%	71%	60%
Correlation	60%	80%	92%	59%	100%	83%	55%
Overlap	60%	72%	84%	59%	86%	71%	65%

^1 ^Correct leave-one-out cross-validation classification rates with gene re-sampling into good and poor outcome groups in 55 tumors

**Table 3:** Classification Rates within Treatment Subgroups^1^

**Gene Set**	**Radiation treated N = 30**	**No Radiation N = 25**	**Chemo treated N = 27**	**No Chemo N = 28**	**Hormone therapy N = 40**	**No Hormone N = 15**
PAM	67%	76%	56%	86%	75%	67%
Correlation	73%	76%	59%	89%	72%	73%
Overlap	67%	72%	59%	79%	75%	60%

^1 ^Correct leave-one-out cross-validation classification rates with gene re-sampling into good and poor outcome groups in 55 tumors
