# Supplementary material for: Identification of a robust gene signature that predicts breast cancer outcome in independent data sets
Source: BMC Cancer. 2007 Apr 11;7:61. doi: 10.1186/1471-2407-7-61 (PMC1855059; doi:10.1186/1471-2407-7-61)
Supplement: Additional File 2 — Excel file containing lowess corrected ratio data. Available under expression ratio values for all cases (and outcome cases) [36]. [file 1471-2407-7-61-S2.doc]

Lowess corrected data files available at: http://cc.ucsf.edu/people/waldman/korkola/outcome.htm
