# Supplementary material for: Identification of a robust gene signature that predicts breast cancer outcome in independent data sets
Source: BMC Cancer. 2007 Apr 11;7:61. doi: 10.1186/1471-2407-7-61 (PMC1855059; doi:10.1186/1471-2407-7-61)
Supplement: Additional File 3 — Excel file containing the median centered, lowess corrected data with print normalization. Available under expression ratio values for all cases after centering and normalization [36]. [file 1471-2407-7-61-S3.doc]

Normalized data files available at http://cc.ucsf.edu/people/waldman/korkola/outcome.htm
