# Supplementary material for: Identification of a robust gene signature that predicts breast cancer outcome in independent data sets
Source: BMC Cancer. 2007 Apr 11;7:61. doi: 10.1186/1471-2407-7-61 (PMC1855059; doi:10.1186/1471-2407-7-61)
Supplement: Additional File 4 — Treeview and cluster files for viewing of treeview images (.cdt, .atr, and .gtr files) Available under hierarchical clustering files [36]. [file 1471-2407-7-61-S4.doc]

Cluster and treeview files available at: http://cc.ucsf.edu/people/waldman/korkola/outcome.htm
