# Supplementary material for: Identification of a robust gene signature that predicts breast cancer outcome in independent data sets
Source: BMC Cancer. 2007 Apr 11;7:61. doi: 10.1186/1471-2407-7-61 (PMC1855059; doi:10.1186/1471-2407-7-61)
Supplement: Additional File 5 — One excel file listing the gene sets derived from PAM, SAM, a correlation based technique, and the overlapping gene list. Available under cDNA clones and gene identifiers in UCSF prediction sets [36]. [file 1471-2407-7-61-S5.doc]

Gene lists file available at http://cc.ucsf.edu/people/waldman/korkola/outcome.htm
